# A comparison of mycotoxin contamination of premium and grocery brands of pelleted cat food in South Africa

**DOI:** 10.4102/jsava.v88i0.1480

**Published:** 2017-11-22

**Authors:** Sanil D. Singh, Sooraj Baijnath, Anil A. Chuturgoon

**Affiliations:** 1 Biomedical Resource Centre, University of KwaZulu-Natal, South Africa; 2 Discipline of Medical Biochemistry, University of KwaZulu-Natal, South Africa

## Abstract

Contamination with mycotoxins is of concern to pet owners and veterinary practitioners owing to their ability to cause disease and exacerbate the pathological changes associated with other diseases. Currently, there is a lack of information regarding the mycotoxin content of common premium brand (PB) and grocery brand (GB) cat feeds. Therefore, we undertook to determine the mycobiota content of feed samples, from both categories (*n* = 6 each), and measured the levels of aflatoxin (AF), fumonisin (FB), ochratoxin A (OTA) and zearalenone (ZEA) by high performance liquid chromatographic analysis. There were high concentrations of mycotoxins in both categories of feed, regardless of the notion that PBs are of a higher quality. The concentration of these toxins may contribute to the development of related pathologies in felines.

## Introduction

Mycotoxins have been implicated in adverse effects in both human and animal health (Fink-Gremmels 1999; Pulina et al. 2014). In a worldwide survey (2004–2011) of over 17 000 samples of feed or feed ingredients, it was found that more than 75% of samples were contaminated by at least one mycotoxin and 40% of the samples contained at least two mycotoxins (Streit et al. 2013). Currently, about 300 mycotoxins have been identified but not all are necessarily implicated in toxicity. The Food and Agriculture Organization (FAO) estimates that a quarter of the food produced globally is contaminated with mycotoxins. This causes significant economic losses as well as poses a serious threat to human and animal health (Bryden 2012; Vasanthi & Bhat 1998). Hence, regulatory limits have been recommended by organisations such as the Food and Drug Administration (FDA) for the common mycotoxins. Mycotoxins commonly implicated in and associated with animal health concerns include aflatoxin (AF), fumonisin (FB), ochratoxin A (OTA), trichothecenes and zearalenone (ZEA) (Boermans & Leung 2007).

Dry, pelleted pet food often contains 5% – 25% of animal protein or its derivatives with the remaining ingredients consisting of corn, corn gluten, wheat, wheat gluten and rice and its by-products, amongst other ‘millings’ (Klich & Pitt 1988). In a highly competitive pet food market, cost-cutting exercises are inevitable, leading to a compromise in the quality of products entering the retail sector. These cereal products that are often unfit for human consumption can act as excellent substrates for fungal proliferation and production of mycotoxins that contribute to liver, kidney and other diseases in pets (Bucci et al. 1998; Dereszynski et al. 2008). It is the contamination of cereals at harvest, post-harvest, manufacture and then storage (Bennett & Klich 2003; Tulpule 1981) that often becomes a health risk to pets by causing mycotoxicosis incidents and death. In 2011, South Africa experienced an outbreak of aflatoxicosis as a result of the consumption of poor quality, low-cost pelleted food (Arnot et al. 2012). The exacerbating factor was mouldy and low-grade peanuts that were contaminated with *Aspergillus flavus* and *Aspergillus parasiticus*.

In this study, we compared the mycotoxin profiles of premium brand (PB) and grocery brand (GB) cat food. PB products are perceived to have low amounts of cereal whilst GBs are perceived to have higher cereal content. Though no major mycotoxin outbreaks have been recorded in felines in recent years, the implication of mycotoxins and their role in feline health cannot be ignored (De Souza & Scussel 2012). Examination of cat food labelling on packaging reveals that claims of high crude protein contents refer largely to vegetable and animal by-products and minimally to meat. Packaging labels provide extensive information on ingredients but limited information on actual percentages of ingredients in the formulation, a trend that is seen in both market segments and often leads to misunderstanding amongst consumers with regard to the nutritional value of the product. Furthermore, information gained from this study may warrant further investigation and contribute to consumer knowledge and feline health.

## Materials and methods

### Materials

Chemicals, reagents and mycotoxin standards were obtained from Merck (South Africa) and Sigma (South Africa) unless otherwise specified. All mycotoxin standards, except the fumonisins, were purchased from Sigma (St. Louis, USA), whilst fumonisin B_1_ (FB_1_) and FB_2_ were purchased from PROMEC (MRC, South Africa). For this study, PB refers to all veterinary restricted brands that may be purchased at veterinary practices or retail veterinary shops (Vetshops) only and generally are priced between R80.00 and R120.00 per kilogram, whilst GBs are commonly sold in supermarket and grocery outlets at a lower price range between R30 and R60 per kilogram.

### Methodology

#### Sampling

Pelleted cat food (*n* = 12) from two marketing channels (PB and GB) were selected for this study. Samples were purchased from their respective outlets in convenient sizes of 2 kg – 3 kg packets. Information on brand, package size, expiry date and barcode serial numbers were recorded. Each packet of food was emptied into a 5-L bucket and thoroughly mixed by shaking. The sampling technique was adapted from methods described by Tittlemier et al. (2011). The bucket was divided into quadrants and approximately 125 g per quadrant sample was scooped up with a clean metal ladle. The samples were thoroughly mixed prior to obtaining a representative sub-sample of 500 g of which a further sub-sample of 200 g was taken by dividing 500 g into four sub-samples and 50 g taken from each quadrant. All feed samples (200 g each) were milled to a fine powder using a mechanical blender (Petron 3600, Germany). The milled samples were used for fungal culture and mycotoxin determination. Remaining samples were resealed and stored in sealed containers at 4 °C until required for further analysis.

**Fungal isolation:** Fungal isolation as well as subculturing and subsequent identification of fungi were done as previously described (Kaufman, Williams & Sumner 1963; Singh & Chuturgoon 2017).

**Mycotoxin extraction and clean-up of feed samples:** Mycotoxin extractions were done as described (Singh & Chuturgoon 2017). Mean recoveries are provided in Table 1.

**TABLE 1:** Mean recoveries of selected mycotoxins after spiking in feed samples using high performance liquid chromatography.

Mycotoxin	Concentration spiked (µg/kg)	Concentration measured (µg/kg)	% of recovery
AFB_1_	100	95.5	95.5
AFB_2_	100	89.0	89.0
OTA	100	94.6	94.6
ZEA	100	93.0	93.0
FB_1_	200	196.4	98.2
FB_2_	200	193.0	96.5

**Thin layer chromatography:** Thin layer chromatography (TLC) was run for each mycotoxin as previously described (Singh & Chuturgoon 2017).

**High performance liquid chromatographic analysis of feed sample extracts:** High performance liquid chromatographic (HPLC) analysis of feed sample extracts was performed as previously described (Singh & Chuturgoon 2017).

## Results

Thin layer chromatography characterisation and HPLC quantitation (μg/mL) were performed for the commonly suspected mycotoxins implicated in pet food contamination, namely, AF, FB, OTA and ZEA (Liggett et al. 1986; Shephard & Sewram 2004; Stenske et al. 2006). The most prevalent fungal isolates in all samples were *Aspergillus* species, *Fusarium* species and *Penicillium* species (Table 2). These fungi were found in both PB and GB feed categories. The fungal species *Aspergillus flavus, Aspergillus fumigatus* and *Aspergillus niger* were more commonly isolated while *A. parasiticus, Aspergillus ochraceus, Aspergillus poae* and *Aspergillus penicillioides* were found less commonly in the samples tested.

**TABLE 2:** Fungal species identification and selected mycotoxin detection in premium brand and grocery brand cat pelleted feed samples.

Fungal species	Fungal sub-species	Fungal isolates (CFU’s/mL)	
		Premium	Grocery
*Aspergillus*	*A. flavus*	***	**
	*A. fumigatus*	*	**
	*A. niger*	**	**
	*A. niveus*	-	-
	*A. ochraceus*	-	**
	*A. parasiticus*	*	**
	*A. penicillioides*	*	*
	*A. poae*	**	-
*Fusarium*	*F. graminearum*	**	***
	*F. verticillioides*	*	*
*Penicillium*	*Penicillium* spp.	**	*
	*P. polonicum*	-	*
	*P. crustosum*	-	*
Other	*Rhizopus* spp.	*+*	-
	Unidentified microbe	*+*	-
	Yeast	*+*	*+*

*A., Aspergillus; F., Fusarium; P., Penicillium*.

* , 100–300 × 10^4^ CFUs;

** , 300–500 × 10^4^ CFUs;

*** , > 500 × 10^4^ CFUs; +, positive only.

Using TLC, all samples in both categories tested positive for four mycotoxins (Table 3). The PB samples appeared to fare worse than GB samples, particularly in terms of AF and ZEA concentrations. HPLC analysis investigated AF for AFB_1_ and AFB_2_ while FB was evaluated for FB_1_ and FB_2_ besides OTA and ZEA. Both PB and GB failed the limits set by the *Fertilizer, Farm Feeds, Agricultural Remedies and Stock remedies Act* (No. 36 of 1947) of 10 ppb (1 ppb = 1 μg/L) for total AFs (South African Government 2009). The levels of AFs (Table 4) detected in the PB were over the set limits for both AFB_1_ (125.02 ppb) and AFB_2_ (11.77 ppb) but GB only exceeded the limits for AFB_1_ (41.57 ppb). The amounts of both AFB_1_ (*p* = 0.0087) and AFB_2_ (*p* = 0.0091) in PB were statistically significantly higher as compared to GB. *Fusarium graminearum* was predominantly isolated in both categories; however, HPLC analysis indicated that the GB had exceedingly high concentrations of both FB_1_ (202.53 ppb) and FB_2_ (118.37 ppb), failing the limit set by the Food and Drug Administration of 100 ppb (FDA 2001). The amounts of both FB_1_ (*p* = 0.028) and FB_2_ (*p* = 0.0041) were significantly higher in GB as compared to PB. OTA (*p* = 0.0196) and ZEA (*p* = 0.0060) levels were significantly higher in PB as compared to GB (Table 4).

**TABLE 3:** The results of thin layer chromatography characterisation.

Mycotoxin	TLC characterisation	
	Premium	Grocery
Aflatoxin	***	**
Fumonisin	*	**
Ochratoxin A	**	**
Zearalenone	**	*

TCL, thin layer chromatography.

*** , very intense spot;

** , intense spot;

* , spot; (intensity of spot as compared to a standard of each mycotoxin).

**TABLE 4:** The results of the high performance liquid chromatographic quantitation of mycotoxins in cat feed extracts.

Feed market segment	AFB_1_	AFB_2_	FB_1_	FB_2_	OTA	ZEA
Premium brand	125.02**	11.77*	9.98	5.45	1.32	9.1*
Grocery brand	41.57	6.30	202.53***	118.37***	0.74	2.27

Note: For statistical significance, data are expressed as mean ± s.d.

OTA, ochratoxin A; ZEA, zearalenone.

* , *p* < 0.05;

** , *p* < 0.005;

*** , *p* < 0.0001

In summary, the PB fared worse than the GB in its AF, OTA and ZEA contamination, whilst GB contained much higher levels of FB than PB.

## Discussion

Cats are obligatory carnivores and require taurine in their diets. A good animal protein source will provide the taurine required for a cat’s good health. The presence of high amounts of mycotoxins in commercial cat diets is indicative of high cereal content. PBs are perceived as better quality with superior nutrition than GBs, but they present a mycotoxin risk due to their high levels of cereal content. A study in Brazil showed a high correlation between diets rich in grains with a reduced immunity to infections in domestic animals (De Souza & Scussel 2012). Clinical signs described for dogs with aflatoxicosis are depression, anorexia, weakness, icterus and sudden death (Arnot et al. 2012; Ketterer et al. 1975; Stenske et al. 2006). Cats with sub-acute aflatoxicosis often show signs of lethargy, anorexia and progressive weight loss. Cats have lower susceptibility to mycotoxicosis than dogs but continuous exposure to low concentrations of mycotoxins in the feed can induce accumulative effects, leading to chronic liver and kidney damage (Dereszynski et al. 2008; Patterson & Roberts 1979).

The PB samples revealed a higher count of *A. flavus* colony forming units (CFUs) than the GB samples. This finding is, however, not surprising as these are ubiquitous soil fungi and common contaminants of corn, groundnuts and other cereal grains used in animal feed production (Leung, Díaz-Llano & Smith 2006) and often implicated in aflatoxicosis. In addition, *Fusarium graminearum* was detected at higher concentrations in the GB samples and are associated with FB, fusaric acid and ZEA production. *Penicillium* species were less commonly noted but PB samples showed higher concentrations than the GB samples. *Penicillium* spp. produces tremorgenic mycotoxins such as roquefortine and penitrem A. These mycotoxins induce tremorgenic mycotoxicosis particularly in canines characterised by acute abdominal pain, salivation, vomiting, fever, muscle tremors with hyperaesthesia, seizures and sometimes even death (Hocking, Holds & Tobin 1988; Naudé et al. 2002; Young et al. 2003). OTA is produced by a number of *Aspergillus* and *Penicillium* species while ZEA is produced by *Fusarium* species (Leung et al. 2006; Shephard & Sewram 2004). OTA and ZEA were detected (by TLC and quantified by HPLC) at very low levels, but various studies have described toxicity at these reported levels (Leung et al. 2006). The role of *Fusarium* mycotoxins in animal health is particularly important economically as they have been implicated in infertility and reproductive dysfunction in sheep, cattle and pigs. Poultry are particularly affected with loss in weight, egg production and gastrointestinal lesions (Antonissen et al. 2014; D’Mello, Placinta & Macdonald 1999; Placinta, D’Mello & Macdonald 1999).

A mycotoxin mix of FB_1,_ FB_2_, OTA and ZEA together with AFs may present a higher risk to illness or mycotoxicosis. Many researchers have reported the simultaneous occurrence of several mycotoxins in feed and feed ingredients (Fox, Hodgkins & Smart 2012; Mwanza 2007; Tulpule 1981). This potent mycotoxin combination may result in synergistic action and potentiate effects that support the multi-aetiological theory (Boermans & Leung 2007; Creppy et al. 2004; Mwanza et al. 2013; Ryu, Jackson & Bullerman 2002).

Irrespective of marketing channels, all products were contaminated with mycotoxins. The mean AF concentration across the various brands indicates that all products failed the prescribed limit (10 ppb; by the *Fertilizer, Farm Feeds, Agricultural Remedies and Stock remedies Act* [No. 36 of 1947]; South African Government 2009). The long-term exposure of cats to mycotoxins may be implicated in numerous clinical conditions such as neoplasia, reduced immunity and poor growth and fertility (De Souza & Scussel 2012).

## Conclusion

PBs are marketed as superior feeds, but their cereal content makes them susceptible to mycotoxin contamination. Many PBs are imported and the higher mycotoxin content may be attributed to lengthy transport in containers on the high seas and high humidity. Though cats may appear to be less susceptible to mycotoxicosis, the risk of long-term exposure to mycotoxins coupled with poor health or concurrent disease could result in increased susceptibility. Further *in vivo* studies are required to evaluate feline susceptibility to mycotoxins.

## References

[CIT0001] Antonissen G., Martel A., Pasmans F., Ducatelle R., Verbrugghe E., Vandenbroucke V. et al., 2014, ‘The impact of Fusarium mycotoxins on human and animal host susceptibility to infectious diseases’, *Toxins* 6, 430–452. 10.3390/toxins6020430 24476707 PMC3942744

[CIT0002] Arnot L.F., Duncan N.M., Coetzer H. & Botha C.J, 2012, ‘An outbreak of canine aflatoxicosis in Gauteng Province, South Africa’, *Journal of the South African Veterinary Association* 83(1), Art. #2, 1–4. 10.4102/jsava.v83i1.2 23327140

[CIT0003] Bennett J. & Klich M, 2003, ‘Mycotoxins’, *Clinical Microbiology Reviews* 16, 497–516. 10.1128/CMR.16.3.497-516.2003 12857779 PMC164220

[CIT0004] Boermans H.J. & Leung M.C, 2007, ‘Mycotoxins and the pet food industry: Toxicological evidence and risk assessment’, *International Journal of Food Microbiology* 119, 95–102. 10.1016/j.ijfoodmicro.2007.07.063 17889389

[CIT0005] Bryden W.L, 2012, ‘Mycotoxin contamination of the feed supply chain: Implications for animal productivity and feed security’, *Animal Feed Science and Technology* 173, 134–158. 10.1016/j.anifeedsci.2011.12.014

[CIT0006] Bucci T.J., Howard P.C., Tolleson W.H., Laborde J.B. & Hansen D.K, 1998, ‘Renal effects of fumonisin mycotoxins in animals’, *Toxicologic Pathology* 26, 160–164. 10.1177/019262339802600119 9502399

[CIT0007] Creppy E.E., Chiarappa P., Baudrimont I., Borracci P., Moukha S. & Carratù M.R, 2004, ‘Synergistic effects of fumonisin B 1 and ochratoxin A: Are in vitro cytotoxicity data predictive of in vivo acute toxicity?’, *Toxicology* 201, 115–123. 10.1016/j.tox.2004.04.008 15297026

[CIT0008] Dereszynski D.M., Center S.A., Randolph J.F., Brooks M.B., Hadden A.G., Palyada K. et al., 2008, ‘Clinical and clinicopathologic features of dogs that consumed foodborne hepatotoxic aflatoxins: 72 cases (2005–2006)’, *Journal of the American Veterinary Medical Association* 232, 1329–1337. 10.2460/javma.232.9.1329 18447777

[CIT0009] De Souza K.K. & Scussel V.M, 2012, ‘Occurrence of dogs and cats diseases records in the veterinary clinics routine in South Brazil and its relationship to mycotoxins’, *International Journal of Applied Science and Technology* 2(8), 129–134.

[CIT0010] D’Mello J., Placinta C. & Macdonald A, 1999, ‘Fusarium mycotoxins: A review of global implications for animal health, welfare and productivity’, *Animal Feed Science and Technology* 80, 183–205. 10.1016/S0377-8401(99)00059-0

[CIT0011] Fink-Gremmels J, 1999, ‘Mycotoxins: Their implications for human and animal health’, *Veterinary Quarterly* 21, 115–120. 10.1080/01652176.1999.9695005 10568000

[CIT0012] Food and Drug Administration (FDA), 2001, *Guidance for industry: Fumonisin levels in human foods and animal feeds*, Food and Drug Administration, Washington, DC.

[CIT0013] Fox M.W., Hodgkins E. & Smart M.E, 2012, *Not fit for a dog!: The truth about manufactured dog and cat food*, Linden Publishing, Fresno, CA.

[CIT0014] Hocking A., Holds K. & Tobin N, 1988, ‘Intoxication by tremorgenic mycotoxin (penitrem A) in a dog’, *Australian Veterinary Journal* 65, 82–85. 10.1111/j.1751-0813.1988.tb07366.x 3401148

[CIT0015] Kaufman D.D., Williams L.E. & Sumner C.B, 1963, ‘Effect of plating medium and incubation temperature on growth of fungi in soil-dilution plates’, *Canadian Journal of Microbiology* 9, 741–751. 10.1139/m63-100

[CIT0016] Ketterer P., Williams E., Blaney B. & Connole M, 1975, ‘Canine aflatoxicosis’, *Australian Veterinary Journal* 51, 355–357. 10.1111/j.1751-0813.1975.tb15946.x 810128

[CIT0017] Klich M. & Pitt J, 1988, ‘Differentiation of *Aspergillus flavus* from *A. parasiticus* and other closely related species’, *Transactions of the British Mycological Society* 91(1), 99–108. 10.1016/S0007-1536(88)80010-X

[CIT0018] Leung M.C., Díaz-Llano G. & Smith T.K, 2006, ‘Mycotoxins in pet food: A review on worldwide prevalence and preventative strategies’, *Journal of Agricultural and Food Chemistry* 54, 9623–9635. 10.1021/jf062363 17177480

[CIT0019] Liggett A., Colvin B., Beaver R. & Wilson D, 1986, ‘Canine aflatoxicosis: A continuing problem’, *Veterinary and Human Toxicology* 28, 428–430. 3776087

[CIT0020] Mwanza M, 2007, ‘An investigation in South African domesticated animals, their products and related health issues with reference to mycotoxins and fungi’, M. Tech thesis, University of Johannesburg.

[CIT0021] Mwanza M., Ndou R.V., Dzoma B., Nyirenda M. & Bakunzi F, 2013, ‘Canine aflatoxicosis outbreak in South Africa (2011): A possible multi-mycotoxins aetiology’, *Journal of the South African Veterinary Association* 84(1), Art. #133, 1–5. 10.4102/jsava.v84i1.133 23905208

[CIT0022] Naudé T., O’Brien O., Rundberget T., Mcgregor A., Roux C. & Flåøyen A, 2002, ‘Tremorgenic neuromycotoxicosis in 2 dogs ascribed to the ingestion of penitrem A and possibly roquefortine in rice contaminated with *Penicillium crustosum*’, *Journal of the South African Veterinary Association* 73, 211–215. 10.4102/jsava.v73i4.589 12665136

[CIT0023] Patterson D. & Roberts B, 1979, ‘Mycotoxins in animal feedstuffs: Sensitive thin layer chromatographic detection of aflatoxin, ochratoxin A, sterigmatocystin, zearalenone, and T-2 toxin’, *Journal of the Association of Official Analytical Chemists* 62, 1265–1267. 521411

[CIT0024] Placinta C., D’Mello J. & Macdonald A, 1999, ‘A review of worldwide contamination of cereal grains and animal feed with Fusarium mycotoxins’, *Animal Feed Science and Technology* 78, 21–37. 10.1016/S0377-8401(98)00278-8

[CIT0025] Pulina G., Battacone G., Brambilla G., Cheli F., Danieli P.P., Masoero F. et al., 2014, ‘An update on the safety of foods of animal origin and feeds’, *Italian Journal of Animal Science* 13, 3571 10.4081/ijas.2014.3571

[CIT0026] Ryu D., Jackson L.S. & Bullerman L.B, 2002, ‘Effects of processing on zearalenone’, *Advances in Experimental Medicine and Biology Series* 504, 205–216. 10.1007/978-1-4615-0629-4_21 11922089

[CIT0027] Shephard G. & Sewram V, 2004, ‘Determination of the mycotoxin fumonisin B1 in maize by reversed-phase thin-layer chromatography: A collaborative study’, *Food Additives and Contaminants* 21, 498–505. 10.1080/02652030410001670175 15204551

[CIT0028] Singh S.D. & Chuturgoon A.A, 2017, ‘A comparative analysis of mycotoxin contamination of supermarket and premium brand pelleted dog food in Durban, South Africa’, *Journal of South African Veterinary Association* 88, a1488 10.4102/jsava.v88i0.1488 PMC6138162 29041787

[CIT0029] South African Government, 2009, *Fertilizers, farm feeds, agricultural remedies and stock remedies act (Avt No.36 of 1947)*, South African Government Gazette No. R227, 2009, March 06, Government Printer, Pretoria.

[CIT0030] Stenske K.A., Smith J.R., Newman S.J., Newman L.B. & Kirk C.A, 2006, ‘Aflatoxicosis in dogs and dealing with suspected contaminated commercial foods’, *Journal of the American Veterinary Medical Association* 228, 1686–1691. 10.2460/javma.228.11.1686 16740069

[CIT0031] Streit E., Naehrer K., Rodrigues I. & Schatzmayr G, 2013, ‘Mycotoxin occurrence in feed and feed raw materials worldwide: Long-term analysis with special focus on Europe and Asia’, *Journal of the Science of Food and Agriculture* 93, 2892–2899. 10.1002/jsfa.6225 23670211

[CIT0032] Tittlemier S., Varga E., Scott P. & Krska R, 2011, ‘Sampling of cereals and cereal-based foods for the determination of ochratoxin A: An overview’, *Food Additives and Contaminants* 28, 775–785. 10.1080/19440049.2011.559278 21623502 PMC3118486

[CIT0033] Tulpule P, 1981, ‘Aflatoxins – Experimental studies’, *Journal of Cancer Research and Clinical Oncology* 99, 137–142. 10.1007/BF00412449 7251631 PMC12253725

[CIT0034] Vasanthi S. & Bhat R.V, 1998, ‘Mycotoxins in foods-occurrence, health & economic significance & food control measures’, *Indian Journal of Medical Research* 108, 212. 9863277

[CIT0035] Young K.L., Villar D., Carson T.L., Ierman P., Moore R.A. & Bottoff M.R, 2003, ‘Tremorgenic mycotoxin intoxication with penitrem A and roquefortine in two dogs’, *Journal of the American Veterinary Medical Association* 222, 52–53, 35. 12523480 10.2460/javma.2003.222.52

